# SPH-DEM approach to numerically simulate the deformation of three-dimensional RBCs in non-uniform capillaries

**DOI:** 10.1186/s12938-016-0256-0

**Published:** 2016-12-28

**Authors:** Hasitha-Nayanajith Polwaththe-Gallage, Suvash C. Saha, Emilie Sauret, Robert Flower, Wijitha Senadeera, YuanTong Gu

**Affiliations:** 1 School of Chemistry, Physics and Mechanical Engineering, Faculty of Science and Engineering, Queensland University of Technology, 2-George Street, Brisbane, QLD 4001 Australia; 2 Research and Development, Australian Red Cross Blood Service, Kelvin Grove, QLD 4059 Australia

**Keywords:** Blood flow, Computational biomechanics, Critical diameter, Discrete element method, Meshfree method, Multiple red blood cells, Smoothed particle hydrodynamics

## Abstract

**Background:**

Blood continuously flows through the blood vessels in the human body. When blood flows through the smallest blood vessels, red blood cells (RBCs) in the blood exhibit various types of motion and deformed shapes. Computational modelling techniques can be used to successfully predict the behaviour of the RBCs in capillaries. In this study, we report the application of a meshfree particle approach to model and predict the motion and deformation of three-dimensional RBCs in capillaries.

**Methods:**

An elastic spring network based on the discrete element method (DEM) is employed to model the three-dimensional RBC membrane. The haemoglobin in the RBC and the plasma in the blood are modelled as smoothed particle hydrodynamics (SPH) particles. For validation purposes, the behaviour of a single RBC in a simple shear flow is examined and compared against experimental results. Then simulations are carried out to predict the behaviour of RBCs in a capillary; (i) the motion of five identical RBCs in a uniform capillary, (ii) the motion of five identical RBCs with different bending stiffness (*K*_*b*_) values in a stenosed capillary, (iii) the motion of three RBCs in a narrow capillary. Finally five identical RBCs are employed to determine the critical diameter of a stenosed capillary.

**Results:**

Validation results showed a good agreement with less than 10% difference. From the above simulations, the following results are obtained; (i) RBCs exhibit different deformation behaviours due to the hydrodynamic interaction between them. (ii) Asymmetrical deformation behaviours of the RBCs are clearly observed when the bending stiffness (*K*_*b*_) of the RBCs is changed. (iii) The model predicts the ability of the RBCs to squeeze through smaller blood vessels. Finally, from the simulations, the critical diameter of the stenosed section to stop the motion of blood flow is predicted.

**Conclusions:**

A three-dimensional spring network model based on DEM in combination with the SPH method is successfully used to model the motion and deformation of RBCs in capillaries. Simulation results reveal that the condition of blood flow stopping depends on the pressure gradient of the capillary and the severity of stenosis of the capillary. In addition, this model is capable of predicting the critical diameter which prevents motion of RBCs for different blood pressures.

## Background

RBCs develop within the bone marrow [1]. When a RBC is initially produced, it contains a nucleus inside the cell. However, RBCs eject their nuclei in the early stages of maturity before entering into the main blood stream [2, 3]. Healthy RBCs exhibit a biconcave discoidal shape with a mean diameter of about 8 µm and a mean thickness of about 2 µm at rest [4]. In the cardiovascular network, blood continuously flows through millions of blood vessels, including smallest blood vessels (capillaries), which are even smaller than the mean diameter of an average healthy RBC. Due to the complex three-dimensional geometric structure of RBCs, they exhibit various types of motion and deformations when they flow in the capillaries [5]. Studying the motion and deformation of RBCs is somewhat difficult due to the micro-dimensions of RBCs and the complexity of blood vessels. In this context, numerical modelling techniques have high potential for explaining and predicting the behaviour of RBCs in the capillaries. Among numerical modelling techniques, recently developed meshfree particle methods (MPM) are most effective for analysing problems with large deformations, such as those associated with RBCs [6]. In particular, smoothed particle hydrodynamics (SPH), one of the popular and well-established meshfree particle approaches, has been used widely by researchers for analysing micro-scale hydrodynamics problems [7, 8]. In addition, spring network models are extensively used to model the elastic membrane of RBCs. However, the combination of these two techniques to model RBC flow has not been properly considered, which is one of the innovations in this study.

Recent developments in computational and numerical techniques have made solving the behaviour of RBCs in three-dimensional domains possible. Imani et al. [9] developed a three-dimensional numerical model to simulate the malaria infected red blood cell (IRBC). In their model, all blood components are modelled by discrete particles, while the malaria parasites inside the RBCs are represented by cluster of rigid particles. For this simulation, the biconcave shaped healthy RBC and the spherical shaped IRBC are used to qualitatively examine the behaviour of RBCs in a narrow 6 µm square channel. Results revealed that IRBC cannot flow through the narrow channels since the IRBCs are stiffer and less deformable [9]. The membrane of the IRBC is modelled by a two-dimensional spring network and the RBCs are considered as two-dimensional disks in a three-dimensional channel. However, the motion and deformation of the RBCs is highly three-dimensional, as the cells exhibit three-dimensional deformations in microvessels [10]. This model was not able to capture the three-dimensional behaviour of the RBCs. Tsubota and Wada [11] proposed a three-dimensional spring network model to estimate the elastic membrane force of a RBC membrane during its tank treading motion. In their model, the RBC membrane is discretised into triangular elements. Assuming a simple shear flow, a small external force was introduced on each node to reproduce the tank treading motion. This model was further improved by Nakamura et al. [12] to simulate the mesoscopic blood flow. However, they assumed that RBCs do not disturb the surrounding flow and a one-way coupling was implemented for the flow-RBC by pre-defining the macroscopic flow field.

Nagayama and Honda [13] developed a three-dimensional model to simulate the behaviour of the RBCs in blood vessels like capillaries the using moving particle semi-implicit (MPS) method. They developed a momentum equation to define the motion of RBCs, considering the inter-particle force, viscous diffusion and external force without solving the Navier–Stokes equations. They studied the motion and deformation of multiple RBCs in bent capillaries. However, this model was not employed to carry out a comprehensive study on motion and deformation of RBCs in capillaries. Pozrikidis [3, 14] developed a three-dimensional model to explain the flow-induced deformation of RBCs. The numerical instabilities of the model and the assumption of axisymmetric behaviour made the model impractical [12]. Recently, the dissipative particle dynamics (DPD) method was employed by Ye et al. [15] to develop a three-dimensional RBC model to predict the flow through a tube containing interacting RBCs. However, they employed only two RBCs with different properties in order to simulate and investigate the effect of the infected RBC on the motion and deformation of the other RBC.

In this paper, a set of (up to five) RBCs is employed to predict the motion and deformation of RBCs more accurately. An advanced numerical modelling technique combining smoothed particle hydrodynamics (SPH) and the discrete element method (DEM) is used to model the motion and deformation of the set of three-dimensional RBCs in microvessels. The RBC membrane is modelled by a three-dimensional spring network using fundamentals of DEM and the RBC membrane is discretised into a finite number of particles. Each particle represents a finite mass with associated with density and pressure. The application of DEM allows to model the larger deformation of the moving RBCs. The forces acting on the RBC membrane are determined based on the minimum energy concept [5, 16–18]. First, we investigate the motion and deformation of five identical RBCs through a stenosed capillary. Then, the effect of RBC membrane bending stiffness (*K*_*b*_) on the motion and deformation of the RBCs in a stenosed capillary is explored. Specifically, this study aims to predict the asymmetric motion and three-dimensional deformation of RBCs, when they have an uncharacteristic membrane bending stiffness due to infection by a disease like malaria. Furthermore, we explore how the motion and deformation behaviour of a set of RBCs in blood vessels with the diameters smaller than those of the blood vessels. Finally, the critical diameter for a stenosed capillary to prevent the motion of RBCs is investigated. With the aid of this model the behaviour of three-dimensional RBCs is predicted, with particular focus on blood flow rate under pathological conditions.

## Numerical model and solution methodologies

### Three dimensional RBC model

**Fig. 1:**
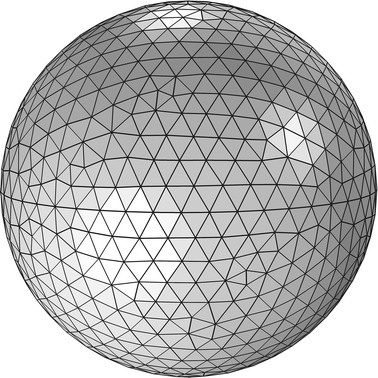
Three-dimensional sphere used to obtain the biconcave shape of the RBC membrane

The membrane of the RBC is modelled by a three dimensional spring network [5]. The RBC membrane is initially assumed to be a sphere with the radius of 3.1 µm. It is discretised into 954 mass points interconnected by 2856 springs as shown in Fig. 1. This number of mass points was chosen such that the minimum distance between two neighbouring points is equal to 0.4 μm, which optimizes the computational cost against the accuracy of the solution [5]. In order to obtain a stable RBC membrane shape, the total energy of the RBC membrane is calculated using the energy functions related to the in plane deformation, bending of the membrane, membrane area and volume constraint. The forces acting on each particle are then calculated based on the principle of virtual work. Finally, the typical discoidal biconcave shape of the RBC membrane is obtained, when the total energy of the membrane is minimized.

The energy generated due to the in-plane deformation, *E*_*S*_ is calculated by:

1 $$E_{S} = \frac{1}{2}K_{S} \sum\limits_{n = 1}^{NS} {\left( {L_{n} - L_{n0} } \right)}^{2}$$

where *NS* is the number of springs, equal to 2856, *K*_*S*_ is the spring constant for stretching/compression, and *L*_*n*_ and *L*_*n0*_ are the present length and original length of the nth spring respectively. Energy associated with the bending deformation, *E*_*B*_ is calculated by:

2 $$E_{B} = \frac{1}{2}K_{B} \sum\limits_{n = 1}^{NB} {L_{n} \tan^{2} \left( {\theta_{n} } \right)}$$

where *NB* is the number of neighbouring triangles, equal to 2856, *K*_*B*_ is the spring constant for bending, and *θ*_*n*_ is the angle between the nth neighbouring triangles. Since the number of lipids per area of the RBC membrane is constant, the membrane area should be conserved locally and as a whole. The energy generated due to the local area changes (*E*_*a*_) and total area changes (*E*_*A*_) are calculated by:

3 $$E_{a} = \frac{1}{2}K_{a} \sum\limits_{n = 1}^{Na} {\left( {\frac{{A_{n} - A_{n0} }}{{A_{n0} }}} \right)}^{2} A_{n0}$$

and

4 $$E_{S} = \frac{1}{2}K_{A} \left( {\frac{{A - A_{0} }}{{A_{0} }}} \right)^{2} A_{0}$$

respectively. Where *A*_*n*_ and *A*_*n0*_ are the current and original areas of the considered triangular element respectively, while *A* and *A*_*0*_ are the current and the reference value of the whole RBC membrane area. Here, *A*_*0*_ is equal to the average area of a healthy matured RBC. *K*_*a*_ and *K*_*A*_ in Eqs. (3) and (4) are the area expansion moduli for local area and whole membrane area respectively. The total enclosed volume by the RBC membrane is conserved and energy generated in the membrane, due to the change in total enclosed volume (*E*_*V*_) is calculated by:

5 $$E_{V} = \frac{1}{2}K_{V} \left( {\frac{{V - V_{0} }}{{V_{0} }}} \right)^{2} V_{0}$$

where *V* and *V*_*0*_ are the current and reference volume of the RBC respectively. The reference volume is equal to the average volume of a healthy matured RBC. Finally, *K*_*V*_ is the penalty coefficient to maintain the *V* as *V*_*0*_.

The total energy (*E*) of the RBC is then calculated by taking the sum of the above energies; *E* = *E*_*S*_ + *E*_*B*_ + *E*_*A*_ + *E*_*a*_ + *E*_*V*_. Finally, the forces acting on each particle are determined based on the principle of virtual work as:

6 $${\mathbf{F}}_{i} = \frac{\partial E}{{\partial {\mathbf{r}}_{i} }}$$

where **F**_*i*_ is the vectorial force acting on the ith particle and **r**_*i*_ is the position vector of the ith particle.

**Fig. 2:**
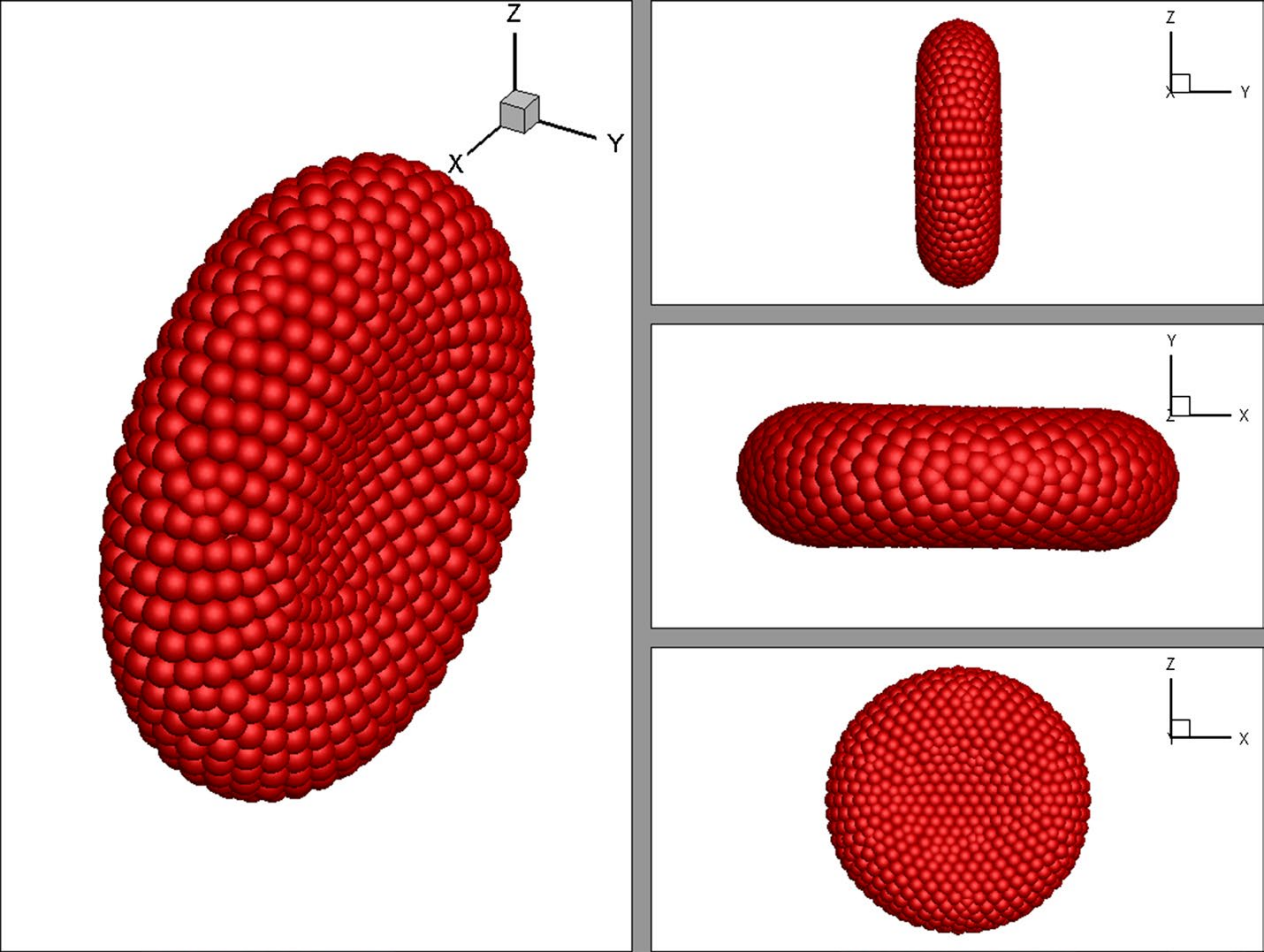
The discoidal biconcave shape of the RBC

In this study the reference volume of the RBC (*V*_*0*_) is set to 60% of the initial volume of the sphere and the reference area of the RBC (*A*_*0*_) is assumed to be the area of the sphere with the radius of 3.1 µm. When the total energy of the RBC is minimised the typical discoidal biconcave shape of a healthy matured RBC is obtained (see Fig. 2).

## Smoothed particle hydrodynamics approach

In the body, RBCs contain haemoglobin and are suspended in plasma. In this study, haemoglobin and plasma components in the problem domain are discretised into a finite number of particles and treated by the smoothed particle hydrodynamics (SPH) method. The Lagrangian form SPH equation for the conservation of momentum,

7 $$\begin{aligned} \frac{{d{\mathbf{v}}_{i} }}{dt} &= - \sum\limits_{j = 1}^{n} {m_{j} \left( {\frac{{p_{j} }}{{\rho_{j}^{2} }} + \frac{{p_{i} }}{{\rho_{i}^{2} }}} \right)} \cdot \nabla_{i} W_{ij} \\ & \quad + \sum\limits_{j = 1}^{n} {m_{j} \frac{{\left( {\mu_{j} + \mu_{i} } \right)\left( {{\mathbf{r}}_{i} - {\mathbf{r}}_{j} } \right) \cdot \nabla_{i} W_{ij} }}{{\rho_{j} \rho_{i} \left| {{\mathbf{r}}_{i} - {\mathbf{r}}_{j} } \right|^{2} }}} \left( {{\mathbf{v}}_{i} - {\mathbf{v}}_{j} } \right) + {\mathbf{F}}_{i}\end{aligned}$$

is used to model the flow field. Here, **v**, *m*, *p*, *ρ* and *µ* are velocity, mass, pressure, density and dynamic viscosity of the SPH particles respectively. In the SPH method, any field function of the *i*th particle is approximated by the same field function values of neighbouring *j*th particles. Finally, **F**_*i*_ and *W* are the external force acting on the particles and the smoothing function.

## Simulation results and discussion

**Fig. 3:**
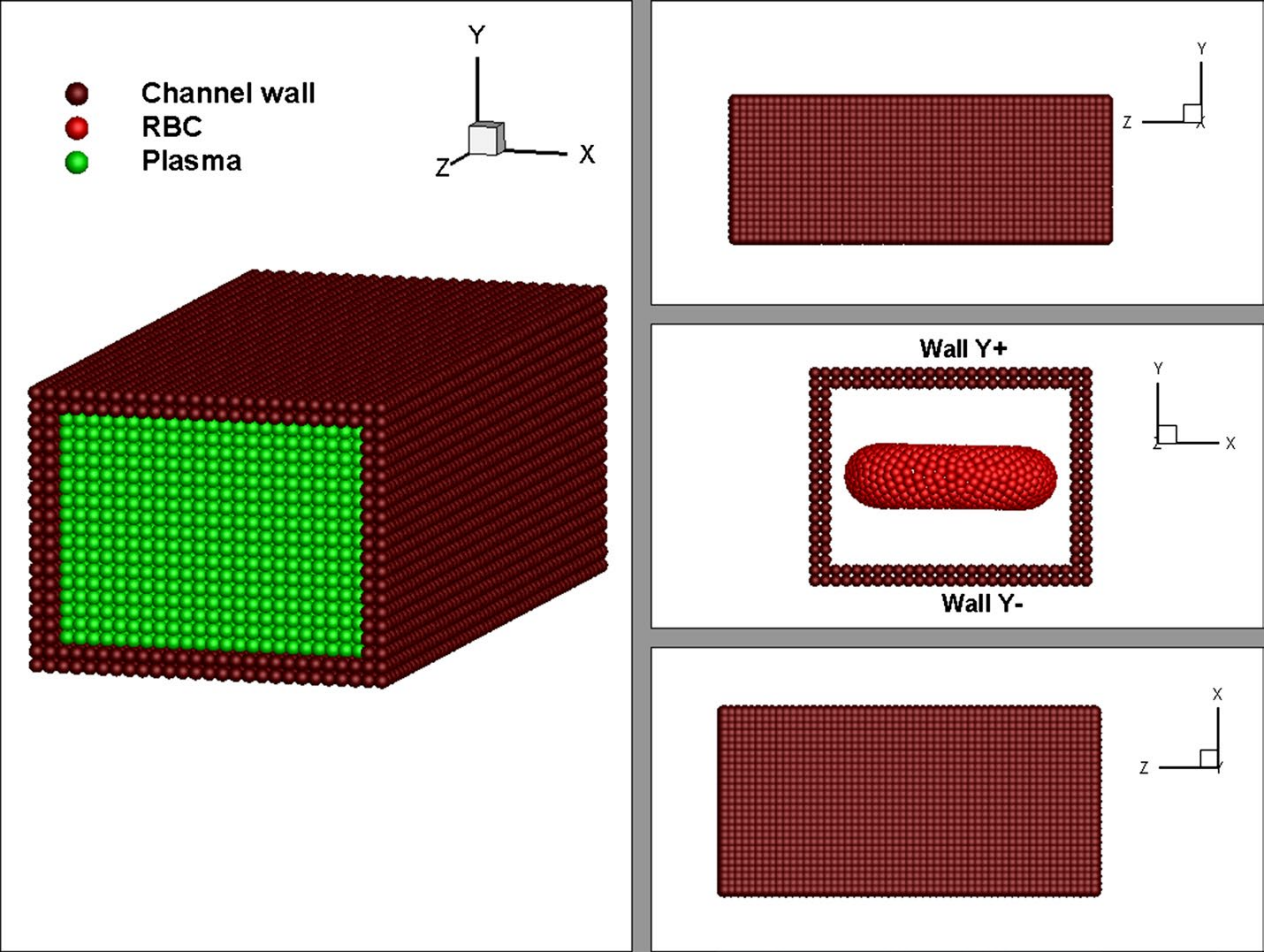
Initial position of the RBC in the rectangular channel

**Fig. 4:**
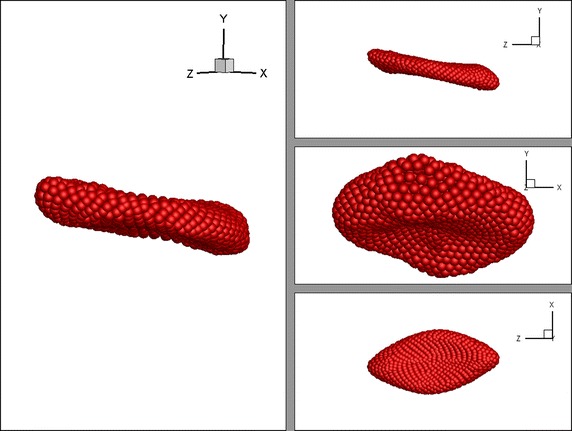
Deformed shape of the RBC under the linear shear flow

The deformation behaviour of the RBC is examined when the RBC is subjected to a linear shear flow. In order to generate the linear shear flow, the RBC is placed into the plasma domain within a rectangular flow channel (see Fig. 3). Then, the top and bottom plates of the rectangular channel are moved at a same constant velocity, **v** but in opposite directions. Periodic boundary conditions are applied to the inlet and the outlet of the channel, such that a particle leaving the outlet will re-enter the fluid domain through the inlet. However, the properties of that particle is recalculated, using the properties of the neighbouring particles at inlet. Due to the motion of the top and bottom plates of the flow channel, plasma particles start to move and generate a pressure acing on the RBC. As a result the RBC elongates and shows a deformed shape (see Fig. 4). The energy constants of the RBC membrane and other parameters are set as in Table 1.

**Table 1:** Key simulation parameters for the model

Parameter	Definition	Value	Reference
*K*_*S*_	Spring constant for stretching/compression	1 × 10^−6^ N/m	[11]
*K*_*B*_	Spring constant for bending	1 × 10^−10^ N	[12]
*K*_*a*_	Area expansion modulus for local area	3 × 10^−3^ N/m	[12]
*K*_*A*_	Area expansion modulus whole membrane area	2 × 10^−3^ N/m	[12]
*K*_*V*_	Penalty coefficient	50 N/m^2^	[12]
*Ρ*_RBC_	Density of the RBC membrane particles	1098 kg/m^3^	[25]
*Ρ*_Cytoplasm_	Density of the cytoplasm particles	1050 kg/m^3^	[26]
*Ρ*_Plasma_	Density of the plasma particles	1025 kg/m^3^	[27]
*µ*_RBC_	RBC membrane viscosity	1 × 10^−3^ Pa s	Set
*µ*_Cytoplasm_	Cytoplasm viscosity	5 × 10^−3^ Pa s	[28]
*µ*_Plasma_	Plasma viscosity	1 × 10^−3^ Pa s	[28]

**Fig. 5:**
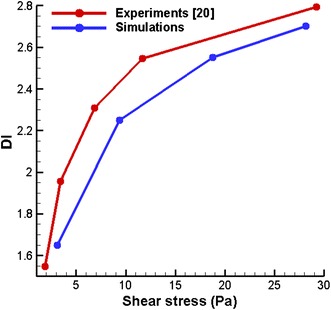
Variation of *DI* of the RBC with the shear stress in a linear shear flow

In this section, the deformation index (*DI)* of the RBC is defined as the ratio between the lengths of the RBC in *z*-direction to *y*-direction and is calculated for different shear stress values. Simulation results reveal that the *DI* increases with the shear stress as shown in Fig. 5. It agrees with the previous experimental results [19] with less than 10% difference. In this study Eq. (8) is used to calculate the shear stress (*τ*),

8 $$\tau = \frac{{2{\mathbf{v}}}}{h}\mu$$

where *µ* and *h* are the dynamic viscosity of the plasma and height of the channel in *y*-direction respectively.

## Deformation behaviour of multiple RBCs through a stenosed capillary

**Fig. 6:**
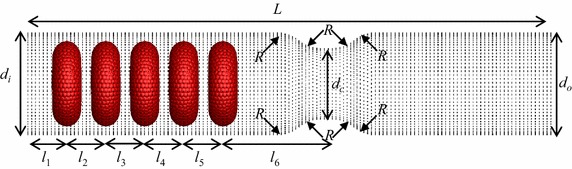
Geometry of the stenosed capillary

In this study, five identical RBCs are used to simulate the motion and deformation behaviour of the RBCs in a stenosed capillary (see Fig. 6). For convenience, the RBC closest to the stenosed section is defined as the 1st RBC (the leading RBC), the RBC closest to the inlet boundary is defined as the 5th RBC (the last RBC); and other RBCs are numbered in order (see Fig. 6). The inlet and outlet diameters (*d*_*i*_ and *d*_*0*_ respectively) are set to 10.0 µm, while the minimum diameter of the stenosed area (*d*_*c*_) is set to 6.8 µm. The total length of the capillary (*L*) is 57.2 µm and the horizontal distance from the 5th RBC’s centre of mass to the inlet boundary (*l*_*1*_) and from the 1st RBC’s centre of mass to the narrowest part of the stenosed (*l*_*6*_) are 3.4 and 10.6 µm respectively (see Fig. 6). The radius of each round corner, *R* is set to 3.2 µm (see Fig. 6). The distances between two consecutive RBCs (*l*_*2*_, *l*_*3*_, *l*_*4*_, and *l*_*5*_) are set to 4 µm. The inlet pressure is set to 1000 Pa, while the outlet pressure is set to zero.

**Fig. 7.**
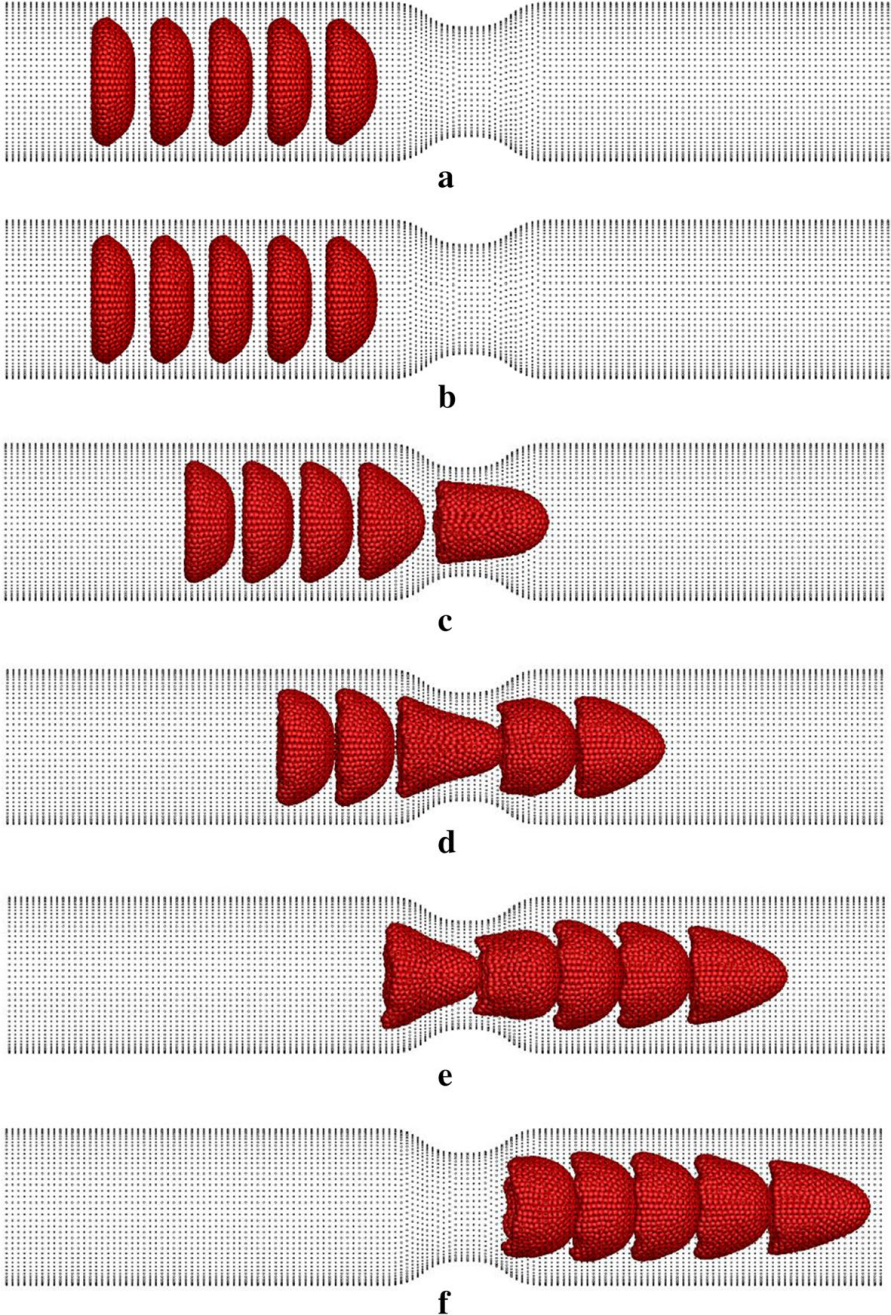
Deformation of five RBCs when they flow in a stenosed capillary with the stenosed diameter of 6.8 µm at **a** t = 0 ms, **b** t = 0.1 ms, **c** t = 0.2 ms, **d** t = 0.3 ms, **e** t = 0.4 ms and **f** t = 0.47 ms

Due to the pressure gradient in the capillary, RBCs begin to flow with plasma and they deform before entering to the stenosed section of the capillary (see Fig. 7b; at *t* = 0.01 ms). However, at this stage, the 1st RBC shows more deformation compared with the following RBCs due to the hydrodynamic interaction between RBCs [20, 21]. The 1st RBC moves though the stenosed section at about *t* = 0.02 ms and it experiences severe deformation during that time (see Fig. 7c). When *t* = 0.025 ms the 1st RBC exits from the stenosed section and it recovers typical deformed parachute shape. When *t* = 0.025 ms, the 2nd RBC moves through the stenosed section and similar to the 1st RBC, the 2nd RBC also undergoes a large deformation. Similar to the 1st and 2nd RBCs, all the RBCs experience severe deformation, when they pass through the narrowest section of the capillary and they recover their typical deformed parachute shape after the stenosed section. Therefore, similar behaviour would be expected for the all the RBCs if they flow further after the stenosed section of the capillary.

**Fig. 8:**
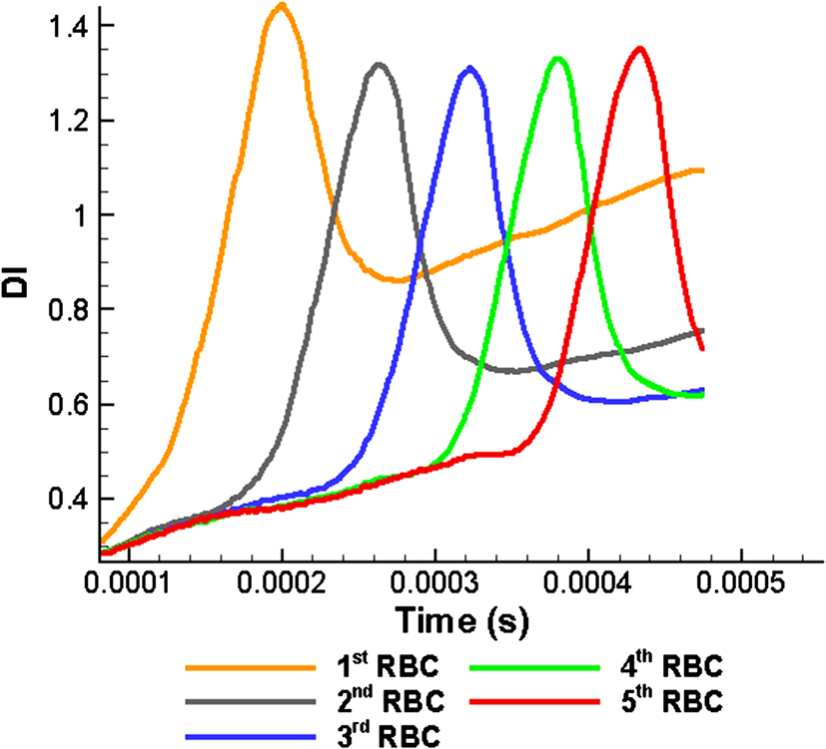
Variation of the *DI* of five RBCs with time when they flow in a stenosed capillary with the stenosed diameter of 6.8 µm

Figure 8 shows the variation of the *DI* of five RBCs with time. As expected, due to the hydrodynamic interaction between RBCs, the 1st RBC shows the maximum *DI* (when *t* = 0.018 ms). The *DI*s of the following RBCs are lesser than that of the 1st RBC when they pass through the stenosed section.

Due to the hydrodynamic interaction between RBCs, a lower *DI* for the 3rd RBC it is expected compared to the 2nd RBC during motion through the stenosed section of the capillary. However, the maximum *DI* of the 3rd RBC does increase compared to the value of the 2nd RBC. Similarly, the 3rd, 4th and 5th RBCs show higher *DIs* compared with their preceding RBC in the stenosed section (see Fig. 8). It is not possible to explain this phenomenon with the aid of the hydrodynamic interaction between RBCs and further studies have to be done to describe this behaviour (this phenomenon will be discussed and explained in next section “Effect of the initial set up of the problem domain on the deformation behaviour of RBCs through a stenosed capillary”. Moreover, the *DI* of the 1st RBC reduces significantly when it exits the stenosed section (see Fig. 8; after *t* = 0.018 ms). However, the *DI* gradually increases again with time (see Fig. 8) and the 1st RBC shows higher *DI* compared with the other RBCs due to the hydrodynamic interaction between RBCs.

**Fig. 9:**
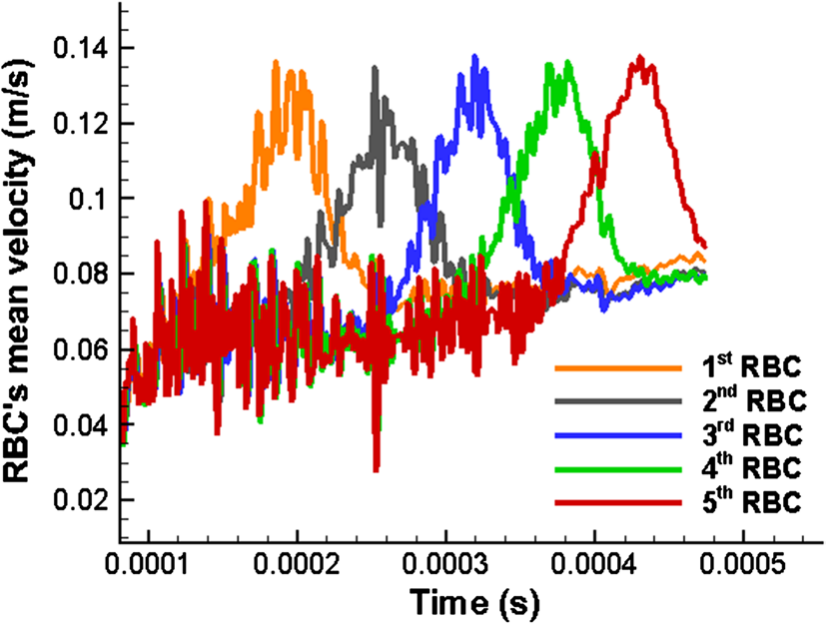
Variation of the mean velocity of five RBCs with time when they flow in a stenosed capillary with the stenosed diameter of 6.8 µm

As can be seen in Fig. 9, a rapid growth in the mean velocities of the RBCs is observed when they flow through the stenosed section of the capillary. All the RBCs flow at almost similar highest mean velocities with only slight variations (see Fig. 9).

## Effect of the initial set up of the problem domain on the deformation behaviour of RBCs through a stenosed capillary

**Fig. 10:**
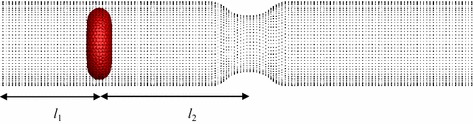
Geometry of the stenosed capillary with a single RBC

In order to explain the increase in the maximum *DI* of the RBCs compared with preceding RBCs when it passing through the stenosed section, further simulations are conducted. In this study, a single RBC is employed with the different horizontal distances from the RBC’s mass centre to the inlet boundary (*l*_*1*_) (see Fig. 10). Consequently the horizontal distances from the RBC’s mass centre to the stenosed section (*l*_*2*_) is changed accordingly. Three cases are studied with *l*_*1*_ = 3.4, 11.4 and 19.4 µm. A capillary with the stenosed diameter of 6.8 µm is used and all the other simulation conditions are kept same as described earlier.

**Fig. 11:**
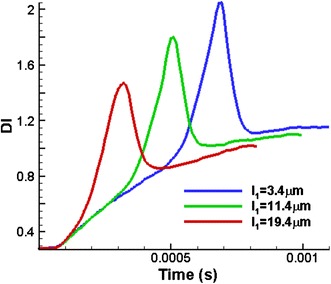
Variation of the *DI* of three RBCs with time when the initial position of the RBC is changed in the capillary with the stenosed diameter of 6.8 µm

The change in the *DI* of the RBCs with time for the three different cases is analysed. The simulation results show that the RBC, initially positioned closer to the stenosed section (*l*_1_ = 19.4 µm) experiences less deformation compared to the other RBCs, initially set farther away from the stenosed section (see Fig. 11). As can be seen in Fig. 11, the RBC relevant to *l*_1_ = 3.4 µm (initially set farther from the stenosed section) undergoes a considerable deformation before it enters to the stenosed section. Moreover, this RBC is subjected to a further deformation while it passes through the stenosed section and it shows the maximum *DI* among the three RBCs. On the other hand, the RBC set closer to the stenosed section (*l*_1_ = 19.4 µm) does not have enough time to deform before entering to the stenosed section. The deformation of this RBC mainly occurs when it passes through the stenosed region. Therefore, it can be concluded that the RBCs set closer to the stenosed section experience less deformation compared with the deformation of the RBCs set farther away from the stenosed section.

In reality, the blood continuously flows and RBCs exhibit deformation (deformed shapes) at all times. However, for the numerical simulations, an initial condition (*t* = 0) is assumed. For the simplicity of this study, it is assumed that the RBC begins with its typical biconcave shape and there is no deformation at *t* = 0. For the above three cases, if the horizontal distance from the RBC’s centre to the stenosed section (*l*_*2*_) increases considerably, the maximum *DI* of all the RBC would have been same, when it passes through the stenosed section. However, it is computationally very expensive to increase the horizontal distance from the RBC’s mass centre to the stenosed section (*l*_*2*_), since it significantly increases the number of particles in the problem domain. The growth in the number of particles in the problem domain would take a longer time to solve the problem and it would be very inefficient. Therefore, the problem domain is controlled for the conditions as explained in earlier sections.

The variation of the maximum *DI* of five RBCs, when they pass though the stenosed capillary, can be explained (see Fig. 8) using the above argument. The 1st RBC of five RBCs shows the highest maximum *DI* when it passes through the stenosed section due to the hydrodynamic interaction between the RBCs. The maximum *DI* of the 2nd RBC is lower than that value of the 1st RBC as expected again due to the hydrodynamic interaction between RBCs. However, the maximum *DI* of the 3rd RBC shows a greater value, compared with the 2nd RBC. This phenomenon happens due to the difference in the horizontal distance from the RBC’s mass centre to the stenosed section (*l*_*2*_). Initially (at *t* = 0) the 3rd RBC is set farther away from the stenosed section compared to the 2nd RBC and the 3rd RBC takes longer time to enter the stenosed section of the capillary. During that time RBC experiences a significant deformation.

The increase in the *DI* of the 3rd RBC just before entering into the stenosed section of the capillary is thus higher than that value of the 2nd RBC. The *DI* of both RBCs increases significantly, while they pass though the stenosed region. However, due to that change in *DI* of the 2nd and 3rd RBCs just before entering the stenosed section of the capillary, the 3rd RBC exhibits higher maximum *DI* when it passes through the stenosed section compared with that value of the 2nd RBC. Similarly, trailing RBCs exhibit higher maximum *DI* compared with their preceding RBCs (except the 1st RBC). Therefore, it can be concluded that the initial horizontal distance from the RBC’s centre to the stenosed section (*l*_*2*_) is a crucial parameter in numerical simulations, when the simulations are carried out to capture the behaviour of RBCs in stenosed capillary. This parameter has to be chosen properly, to obtain reliable enough results without affecting the computation cost too much. However, this study was limited to the conditions discussed for Fig. 7.

## Deformation behaviour of the RBCs with different bending stiffness values in a stenosed capillary

In this study, the effect of RBC membrane bending stiffness on motion and deformation is studied. Five identical RBCs are used to simulate the motion and deformation behaviour of the RBCs in a stenosed capillary. A capillary with a total length of (*L*) of 57.2 µm is used for this study. The inlet (*d*_*i*_) and outlet diameters (*d*_*0*_) of the capillary are set to 10.0 µm. In this study the severity of the stenosed section is further increased in order to clearly compare the effect of the membrane bending stiffness of the RBC. The diameter of the stenosed area (*d*_*c*_) is set to 5.2 µm. The inlet pressure is set to 500 Pa, while the outlet pressure is set to zero. The membrane bending stiffness of the RBCs changes when they are infected by diseases like malaria and cancers. In order to investigate the behaviour of diseased RBCs, the membrane bending stiffness of all the RBCs is changed from the typical value, *K*_*b*_ (1 × 10^−10^ N) to 0.1 *K*_*b*_, 10 *K*_*b*_, 20 *K*_*b*_, 30 *K*_*b*_ and 40 *K*_*b*_. The five identical RBCs and all the other simulation parameters are set as described earlier.

**Fig. 12:**
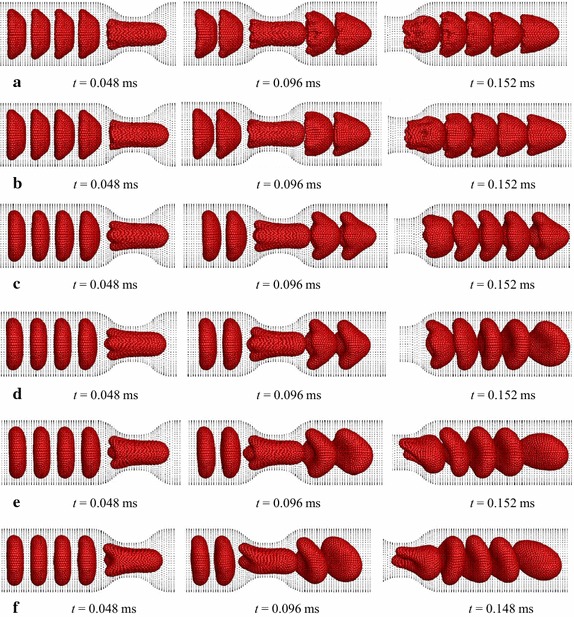
Deformation of five identical RBCs with the membrane bending stiffness of **a** 0.1 *K*_*b*_, **b** *K*_*b*_, **c** 10 *K*_*b*_, **d** 20 *K*_*b*_, **e** 30 *K*_*b*_ and **f** 40 *K*_*b*_ when they flow in a stenosed capillary with the stenosed diameter of 5.2 µm (*K*_*b*_ = 1 × 10^−10^ N)

**Fig. 13:**
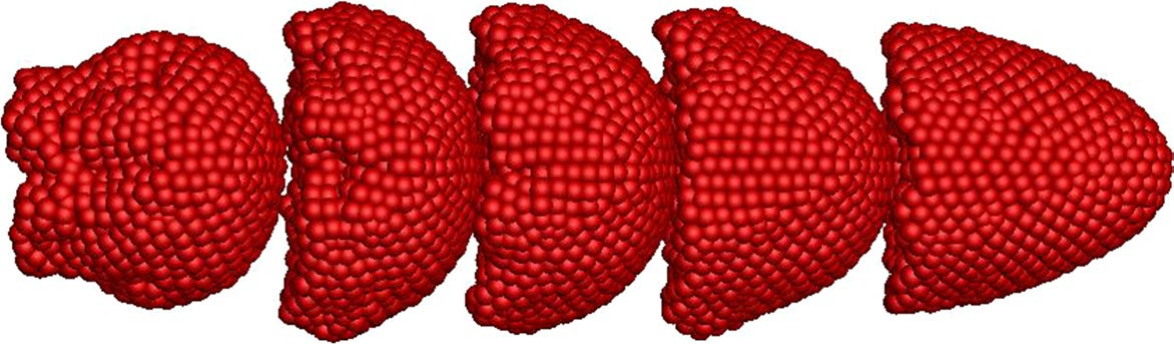
The deformed shapes of the five identical RBCs with the membrane bending stiffness of 0.1 *K*_*b*_ at *t* = 0.148 ms

Simulation results reveal that the RBCs show nearly fully symmetrical deformed shapes (see Fig. 12a) throughout their motion in the stenosed capillary when the membrane bending stiffness of the RBCs is decreased by ten times (0.1 *K*_*b*_). However, they show local uneven deformation with wrinkles on the membrane (see Figs. 12a, 13) when they pass through the stenosed region and just after the stenosed section. On the other hand, the five RBCs with higher bending stiffness values do not show any wrinkles on the deformed membrane. However, the 1st RBC of five RBCs with bending stiffness of 10 *K*_*b*_ shows some asymmetric behaviour compared to the other four RBCs when it reaches the downstream of the capillary (see Fig. 12c at *t* = 0.152 ms). The asymmetric behaviour of that RBC is clearly evidenced when the RBCs have higher bending stiffness values; 20, 30 and 40 *K*_*b*_ (see Fig. 12d–f at *t* = 0.152 ms). Interestingly, the RBCs with the highest bending stiffness values (30 and 40 *K*_*b*_) do not show observable deformation during their motion before the stenosed section (see Fig. 12e, f at *t* = 0.048 ms). However, they deform into bullet-like shapes when they pass through the stenosed region and the deformed RBCs are not symmetrical in shape (see Fig. 12e, f at *t* = 0.096 ms). Furthermore, the RBCs show rolling motions after the stenosed region of the capillary (see Fig. 12e, f at *t* = 0.152 ms). Additionally, the RBCs with the highest bending stiffness value (40 *K*_*b*_) exhibit complicated asymmetric deformed shapes when passing through the narrowest section of the capillary.

**Fig. 14:**
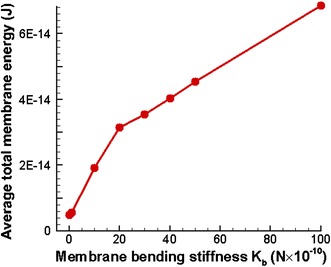
Variation of the average total membrane energy of a single RBC with the membrane bending stiffness, when five identical RBCs flow in a stenosed capillary with the stenosed diameter of 5.2 µm (average is calculated using the maximum total membrane energy of five RBCs)

The RBCs are subject to the largest deformation when they pass through the stenosed section of the capillary. As a result of the deformation, the total membrane energy of the RBCs reaches a maximum value when they pass through the stenosed section of the capillary. The average total membrane energy of a RBC is calculated when it passes through the stenosed section and plotted against the membrane bending stiffness (*K*_*b*_) value of the RBC as shown in Fig. 14. It can be seen from Fig. 14 that the average total membrane energy of one RBC increases significantly with the membrane bending stiffness (*K*_*b*_) of the RBCs, since *K*_*b*_ is dominant on the total energy of the RBCs. Furthermore, the RBCs with higher membrane energies are very unstable in terms of energy and they are vulnerable to rupture [22].

It is not possible to employ the *DI* to compare the amount of deformation, when RBCs show highly asymmetrical three-dimensional deformed shape. Furthermore, the average total membrane energy does not reflect the amount of bending or the *DI* of the RBCs, since the higher membrane bending stiffness values always contribute for higher total membrane energy of the RBCs. Therefore, the average membrane energy (*E*_*b*_) is normalized using membrane bending stiffness (*K*_*b*_). Recalling Eq. (2), this *E*_*b*_/*K*_*b*_ parameter expresses the amount of deformation or the deformability of the RBC. Therefore this parameter can be used to compare the deformability of RBCs, when they have different membrane bending stiffness values.

9 $$\frac{{E_{b} }}{{K_{b} }} = \frac{1}{2}\sum\limits_{n = 1}^{NB} {L_{n} \tan^{2} \left( {\frac{{\theta_{n} - \theta_{n,0} }}{2}} \right)}$$

**Fig. 15:**
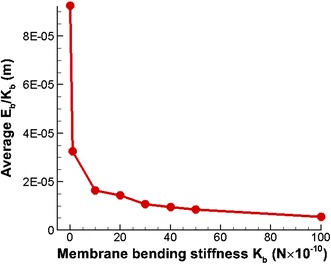
Variation of the average *E*_*b*_/*K*_*b*_ with membrane bending stiffness when five identical RBCs flow in a stenosed capillary with the stenosed diameter of 5.2 µm

In this study, *E*_*b*_/*K*_*b*_ is employed to compare the amount of deformation of the RBCs. It can be seen in Fig. 15, that the RBC with the lowest membrane stiffness (0.1 *K*_*b*_) shows a very high *E*_*b*_/*K*_*b*_ value compared to RBCs with the typical membrane bending stiffness (*K*_*b*_). The *E*_*b*_/*K*_*b*_ ratio decreases substantially when the membrane bending stiffness of the RBCs increases from *K*_*b*_ to 10 *K*_*b*_ (see Fig. 15). Moreover, *E*_*b*_/*K*_*b*_ further decreases gradually with the membrane bending stiffness of the RBC when the membrane bending stiffness of the RBCs increases from 10 to 50 *K*_*b*_ (see Fig. 15). However, that value reaches a more or less steady value, when the membrane bending stiffness increases from 50 to 100 *K*_*b*_. Therefore, irrespective of the membrane bending stiffness of the RBCs, RBCs deform a certain amount in order to pass through the stenosed section of the capillary. It can be concluded that the *DI* or the amount of deformation of the RBCs is governed by the diameter of the stenosed section. Moreover, in order to flow the blood through the cardiovascular network, all the RBCs deform and squeeze through stenosed sections, depending on the diameter of the stenosed section.

## Deformation behaviour in narrow capillaries

**Fig. 16:**
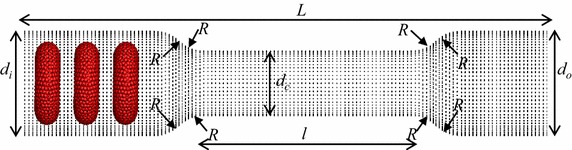
The geometry of the capillary with narrow section

It is generally known that the diameter of capillaries varies between 5 and 10 µm [23]. In this section, the motion and deformation of three RBCs are investigated in capillaries with even narrower sections, with diameters less than RBCs at rest. Three RBCs with identical properties are employed for this simulation in a capillary with the total length (*L*) of 60.0 µm and the length of the narrow section (*l*) of the capillary is set to 21.2 µm (see Fig. 16). The diameter of the narrow section (*d*_*c*_) is set to 6.0 µm, in order to represent a lung capillary. The radius of each round corner, *R* is set to 3.5 µm (see Fig. 16). The inlet (*d*_*i*_) and outlet (*d*_*i*_) diameters of the capillary is set to 10.0 µm as can be seen in Fig. 16, while the inlet and outlet pressures are set to 1000 Pa and zero respectively.

**Fig. 17:**
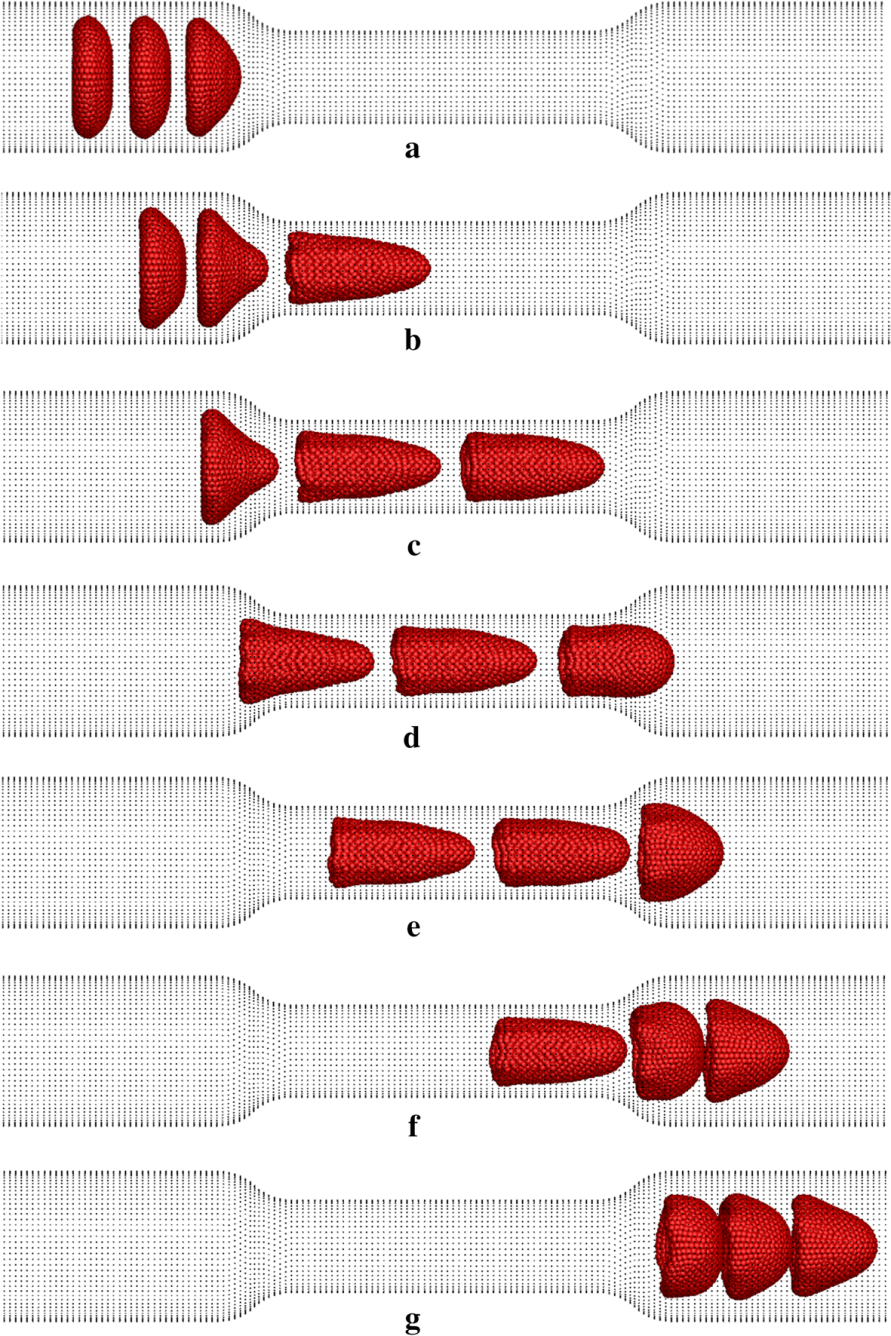
Deformation of three RBCs when they flow in a narrow capillary with the narrow section’s diameter of 6.0 µm at **a** *t* = 0.28 ms, **b** *t* = 0.72 ms, **c** *t* = 1.12 ms, **d** *t* = 1.36 ms, **e** *t* = 1.60 ms, **f** *t* = 1.96 ms and **g** *t* = 2.44 ms

**Fig. 18:**
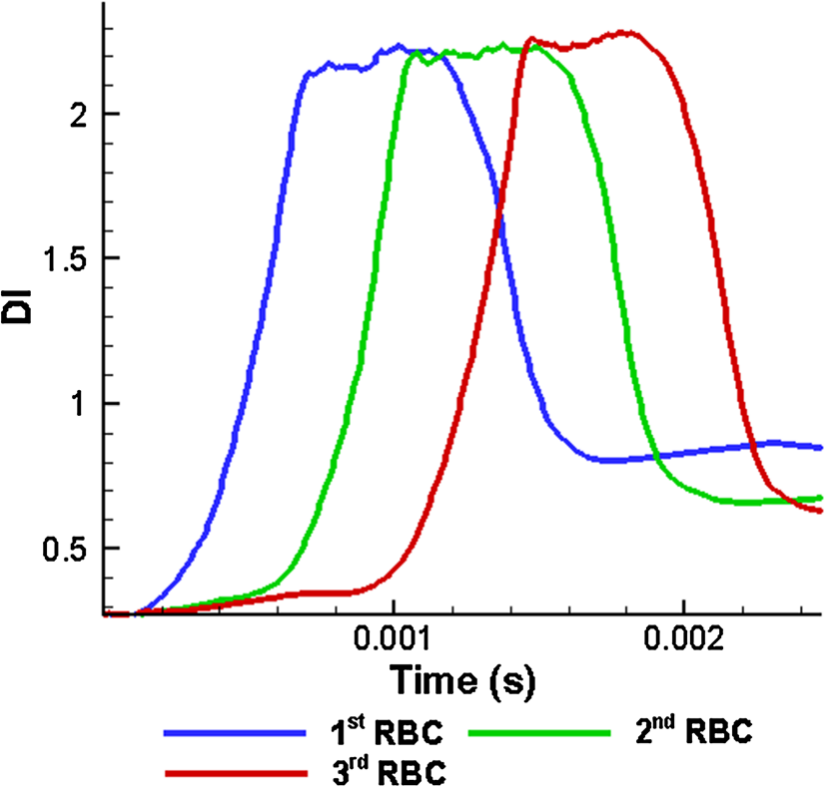
Variation of the *DI* of three RBCs with time when they flow in a capillary with a narrow section

Simulation results shown in Fig. 17 show that three RBCs initially deform into the parachute shapes before entering to the narrow section of the capillary. Interestingly, they exhibit bullet-like shapes when they flow through the narrow section of the capillary (see Fig. 17) while the bullet-like deformed shapes of the RBCs remain unchanged during their whole motion in the narrower section of the capillary. During that time the *DI*s of the three RBCs are similar to each other (see Figs. 17, 18). However, after the narrow section, the 1st RBC exhibits a more deformed shape with a higher *DI,* while the 3rd RBC shows a rounder shape with a lower *DI* due to the hydrodynamic interaction between the RBCs. On the other hand, the 2nd RBC takes an intermediate *DI* compared with the 1st and the 3rd RBCs. This difference in the *DI*s of the RBCs occurs due to the hydrodynamic interaction between the RBCs.

**Fig. 19:**
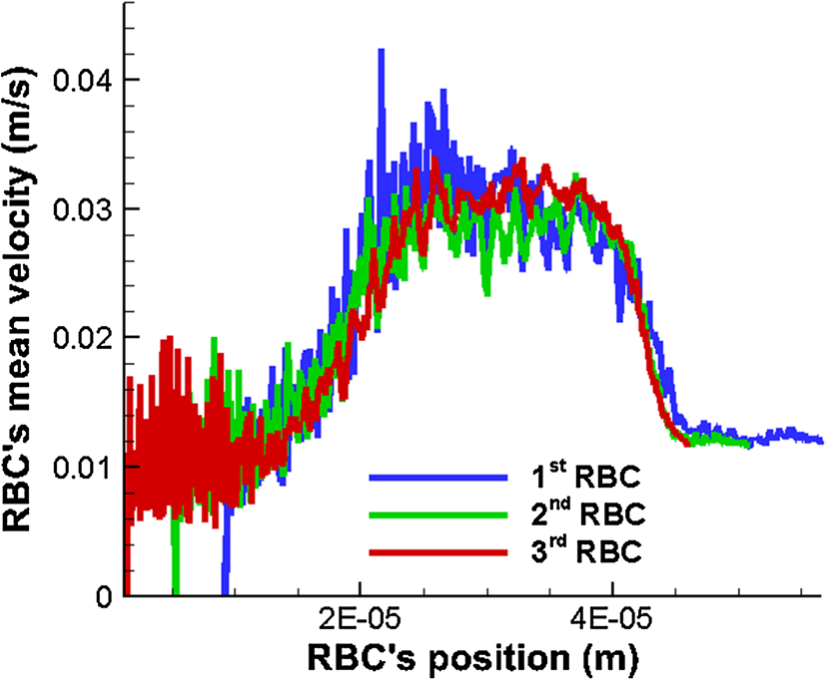
Variation of the mean velocity of three RBCs with their position in the capillary when they flow in a capillary with a narrow section

The mean velocities of the three RBCs gradually increase before the narrow section of the capillary (see Fig. 19). Three RBCs reach maximum steady mean velocities, when they flow through the narrow section. However, the mean velocities of the three RBCs drop back to lower values after the narrow section of the capillary. Initially, the mean velocity curves show very high fluctuations which reflects the unstable nature of the RBCs under theses simulation conditions. However, as can be seen from Fig. 18, the mean velocities of the three RBCs show stable values with less fluctuations when they leave the narrow section of the capillary. Therefore, it can be concluded that the RBCs have reached a stable shape in terms of energy and the particles used to represent the RBCs’ membrane do not move in order to minimise the membrane energy.

## Critical diameter of the stenosed section to stop the motion of blood flow

The blood flow rate in a capillary reduces when the capillary has a stenosed section and it causes to reduce the overall blood rate through the cardiovascular network. Depending on the severity of the stenosis, there is a high risk of microvascular blockage, which may lead to completely stoppage of the blood flow in that capillary [24]. In this section, the critical stenosed diameter of a capillary is investigated to halt the blood flow in the capillary with the aid of five identical RBCs. In this study the diameter of the stenosed section is changed to 8.4, 7.6, 6.8, 6.0, 5.2, 4.4, 3.6 and 2.8 µm in the capillary and the radius of each round corner, *R* is adjusted accordingly. The inlet (*d*_*i*_) and outlet diameters (*d*_*0*_) of the capillary are set to 10.0 µm, while the total length of the capillary (*L*) is set to 57.2 µm. The inlet and outlet pressures are set to 1000 Pa and zero respectively.

**Fig. 20:**
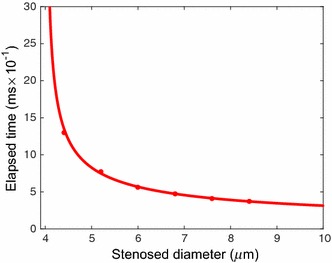
Variation of the time taken by the 1st RBC to reach the outlet of the capillary with different stenosed diameter values, when the inlet and outlet pressure are 1000 Pa and 0 respectively

The time taken by the 1st RBC to reach the outlet of the capillary is measured for each case. When the stenosed diameter of the capillary is 3.6 and 2.8 µm, no motion of the five RBCs is observed. However, the five RBCs start to flow very slowly in the capillary when the stenosed diameter of the capillary is 4.4 µm. As can be seen in Fig. 20, the time taken by the 1st RBC to reach the outlet of the capillary is about 1.3 ms and that is the slowest among all the other cases. Furthermore, when the stenosed diameter of the capillary is changed from 4.4 to 5.2 µm, the time taken by the 1st RBC to reach the outlet of the capillary reduces significantly. Further increase in the stenosed diameter of the capillary reduces the elapsed time more. However, as can be seen from Fig. 20, the time taken by the 1st RBC to reach the outlet of the capillary reaches a nearly stable value when the stenosed diameter of the capillary increases further. With the aid of the Matlab curve fitting tool, the stenosed diameter of the capillary is predicted when the elapsed time tends to infinity. It is found from Fig. 20 that the time taken by the 1st RBC to reach the outlet of the capillary tends to infinity when the diameter of the stenosed section of the capillary is 4.004 µm. Hence, it can be concluded that the five RBCs do not show any motion in a stenosed capillary with inlet pressure of 1000 Pa and outlet pressure of zero when the minimum diameter of the stenosed section 4.004 µm. Therefore, 4.004 µm is the critical diameter for the stenosed capillary, which stops the blood flow for the above inlet and outlet pressures.

However, in reality, when the blood flow stops, the blood pressure builds up before the stenosed section. In order to investigate the effect on the pressure, the simulations are carried out for different inlet pressure values. Similar to the previous case, the critical diameter of the stenosed section of the capillary, which stops the blood flow, is found for different inlet pressure values. In this study the inlet pressure of the stenosed capillary is changed to 500, 1000, 1500, 2000 and 2500 Pa while keeping the outlet pressure of the capillary to zero. All the other parameters are kept constant.

**Fig. 21:**
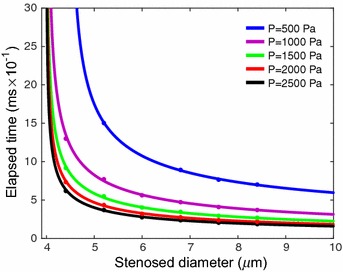
Variation of the time taken by the 1st RBC to reach the outlet of the capillary with different stenosed diameter values, for different inlet pressure values while the outlet pressure is zero

Initially, the inlet pressure is set to 500 Pa and the diameter of the stenosed section of the capillary is changed to 8.4, 7.6, 6.8, 6.0, 5.2, 4.4, 3.6 and 2.8 µm. Then, the time taken by the 1st RBC to reach the outlet of the capillary is measured. Similar to the previous case, time taken by the 1st RBC to reach the outlet of the capillary increases when the stenosed diameter of the capillary decreases. However, the predicted critical diameter of the capillary with the inlet pressure of 500 Pa is 4.472 µm, which is higher than the predicted critical diameter (4.004 µm) of the capillary with the inlet pressure of 1000 Pa. However, when the inlet pressure of the capillary increases (i.e. 1500, 2000 and 2500 Pa), the critical diameter for the stenosed capillary, which stops the blood flow do not show significant variation (see Fig. 21; Table 2) compared with the predicted critical diameter of the capillary with the inlet pressure of 1000 Pa. (see Fig. 21; Table 2).

**Table 2:** The critical diameter of the stenosed section of the capillary for different inlet pressures

Inlet pressure of the stenosed capillary (Pa)	Critical diameter of the stenosed section (µm)
500	4.472
1000	4.004
1500	3.985
2000	3.992
2500	3.989

## Conclusions

A three-dimensional spring network model based on DEM is used in combination with the SPH method to model the motion and deformation of RBCs in capillaries. From this study, the below conclusions are drawn.

In numerical simulation, initial setting of the RBCs directly affects the deformation behaviour of the RBCs. The lengths of the capillaries should be long enough to obtain reliable enough results without affecting the computation cost too much.

When the membrane bending stiffness of the RBCs increase like in malaria infected RBCs they show highly asymmetrical deformed shapes and rolling motions. On the other hand, the RBCs with lower membrane bending stiffness values exhibit wrinkles on the membrane when they are deforming.

Irrespective of the membrane bending stiffness of the RBCs, RBCs deform a certain amount in order to pass through the stenosed section of the capillary.

The RBCs exhibit bullet-like shapes when they flow through the capillaries with narrower sections, which are narrower than the diameter of the RBCs at rest. However, they show parachute shapes when the diameter of the section they are moving through, increases.

There is a certain critical diameter for a given stenosed capillary and for a given pressure gradient which completely stops the motion of blood with RBCs, which leads to microvascular blockages.
